# A Payment Incentive to Improve Confirmatory Testing in Men With Prostate Cancer

**DOI:** 10.1001/jamanetworkopen.2025.30624

**Published:** 2025-09-05

**Authors:** Arnav Srivastava, Samuel R. Kaufman, Addison Shay, Mary Oerline, Xiu Liu, Preeti Chachlani, Paula Guro, Dawson Hill, Monica Van Til, Susan Linsell, Corinne Labardee, Christopher Dall, Kassem S. Faraj, Avinash Maganty, Khurshid R. Ghani, Tudor Borza, Kevin B. Ginsburg, Brent K. Hollenbeck, Vahakn B. Shahinian

**Affiliations:** 1 Dow Division of Health Services Research, Department of Urology, University of Michigan, Ann Arbor; 2 Department of Urology, Massachusetts General Hospital, Boston; 3 Department of Urology, Wayne State University School of Medicine, Detroit, Michigan

## Abstract

**Importance:**

Among men with favorable-risk (ie, low-risk or favorable intermediate-risk) prostate cancer, confirmatory testing substantially improves the detection of aggressive cancers that may merit treatment instead of conservative management. Despite guideline recommendations, confirmatory testing is inconsistently used, and more than half of men do not receive it. Value-based interventions and payment incentives may improve care quality by motivating adherence to guideline-concordant care.

**Objective:**

To examine the use of confirmatory testing among men with low-risk prostate cancer, after the application of a multifaceted intervention, which included physician education and a payment incentive, sponsored by a commercial payer to support its use.

**Design, Setting, and Participants:**

This cohort study used data from the Michigan Urological Surgery Improvement Collaborative on men who received a diagnosis of low-risk prostate cancer between January 1, 2017, and July 1, 2022, with a minimum 6 months of follow-up. Statistical analysis was performed from October 2024 to June 2025.

**Exposure:**

Multifaceted intervention with a payment incentive, applied specifically to men who received a diagnosis of low-risk prostate cancer between April 1, 2018, and May 30, 2019. On meeting the payment incentive’s benchmark (ie, ≥45% of men with low-risk prostate cancer complete confirmatory testing within 6 months of diagnosis), the insurer would distribute enhanced reimbursement on claims covered by commercial preferred provider organization plans.

**Main Outcomes and Measures:**

Confirmatory testing completion (ie, magnetic resonance imaging before or after diagnostic biopsy, repeat prostate biopsy, or genomics test) relative to the preincentive period among men with low-risk prostate cancer. Secondary analyses examined practices by baseline confirmatory testing completion and proportion of patients with insurance plans covered by the insurer sponsoring the payment incentive.

**Results:**

The study included 6609 patients (median age, 65 years [IQR, 60-70 years]), of whom 72.9% (n = 4818) elected for active surveillance. Confirmatory testing increased between 2017 (44.6% [725 of 1625]) and 2022 (64.3% [774 of 1203]) (*P* < .001). During the payment incentive period, patients had a 7.5% (95% CI, 0.0%-15.4%; *P* = .06) increase in the predicted probability of confirmatory testing completion relative to the preincentive period, although this change was not statistically significant (odds ratio, 1.43 [95% CI, 0.99-2.09]; *P* = .06).

**Conclusions and Relevance:**

In this cohort study of men with prostate cancer, confirmatory testing completion improved over the study period. However, the payment incentive was not associated with a robust increase in its use. The results suggest collaboration between payers and physicians has the potential to improve measures of prostate cancer care quality, but also highlight the challenges associated with payment incentives and alternative payment model implementation.

## Key Points

**Question:**

Was a payment incentive, as part of a multifaceted intervention, associated with an increase in confirmatory testing completion (magnetic resonance imaging, repeat prostate biopsy, or genomics test) among men with low-risk prostate cancer?

**Findings:**

In a cohort study of 6609 patients, confirmatory testing completion increased over the study period, from 2017 to 2022. Patients who received the incentive had a 7.5% increase in predicted probability of confirmatory testing relative to the preincentive period, but this finding was not statistically significant.

**Meaning:**

This study suggests that the payment incentive was not associated with a robust increase in confirmatory testing, highlighting the potential as well as the challenges of alternative payment model development.

## Teaser

This cohort study uses data from the Michigan Urological Surgery Improvement Collaborative to examine the completion rate of confirmatory testing among men with low-risk prostate cancer after the application of a multifaceted intervention that included a payment incentive.

## Introduction

Most men with less-aggressive prostate cancer (ie, low-risk or favorable intermediate-risk disease) do not require immediate treatment and can be appropriately offered conservative management.^1^ However, over one-third of these patients may harbor more aggressive disease not found on initial biopsy.^2,3,4,5,6^ To mitigate concerns of risk misclassification, clinical guidelines recommend completion of confirmatory testing—magnetic resonance imaging (MRI), repeat prostate biopsy, or genomic testing—within 6 to 12 months of the diagnostic biopsy.^7,8^ Confirmatory testing allows for the detection of more aggressive cancers that may merit treatment as opposed to conservative management. Despite these guidelines and the importance of confirmatory testing, it is inconsistently used.^9,10,11,12^ For example, in recent analyses of Medicare claims and large registry data, between 40% and 60% of men miss this critical measure of prostate cancer care quality.^13,14,15^

Disease-specific payment models represent an opportunity for insurance payers to leverage reimbursement policy to improve quality and directly engage specialty care physicians.^16^ In some specialty care contexts, such as ventral hernia repair, payers have offered increased remuneration for physician behaviors aligned with quality, such as documentation thoroughness.^17^ Largely, however, most pay-for-performance initiatives and national payment models (eg, accountable care organizations) are primary care–centric, emphasizing care coordination and reduction of low-value health care.^18^ These broad model designs have had a modest effect on care delivered by urologists.^19^ Consequently, in the development of alternative payment models there remains a gap in performance-based incentives that directly engage urologists, even for commonly treated conditions such as prostate cancer.

We evaluated a payment incentive in the Michigan Urological Surgery Improvement Collaborative (MUSIC), sponsored by Blue Cross Blue Shield of Michigan (BCBSM), supporting the completion of confirmatory testing within 6 months of diagnosis among men with low-risk prostate cancer. The incentive was part of a multifaceted intervention, which included physician education and audit and feedback reporting, to improve confirmatory testing within the collaborative. We hypothesized that the intervention would be associated with an acceleration of confirmatory testing completion during its measurement period. Furthermore, we postulated that this association would be magnified among the urology practices with the lowest use of confirmatory testing (ie, most room to improve) and the largest proportion of patients covered by BCBSM (ie, the practices standing to benefit the most from the payment incentive). Last, we hypothesized that, while the intervention targeted men with low-risk prostate cancer, these efforts to increase confirmatory testing would spill over and improve testing rates among men with low-volume favorable intermediate-risk disease.

## Methods

### Study Population

We identified patients from MUSIC, a quality improvement collaborative that captures 48 urology practices and over 90% of urologists within Michigan.^20^ All practices and institutions obtained prior approval or exemption for participation in MUSIC. The University of Michigan Institutional Review Board deemed this analysis as exempt and informed consent was not required because the data were deidentified. Our results reporting adhered to the Strengthening the Reporting of Observational Studies in Epidemiology (STROBE) reporting guideline.

From the MUSIC registry, we identified men with low-risk (grade group 1) and low-volume favorable intermediate-risk (grade group 2 with ≤3 positive cores and ≤50% greatest individual core involvement) who received a diagnosis of prostate cancer between January 1, 2017, and July 1, 2022. Only men with at least 6 months of follow-up after diagnosis were included.

Patient race and ethnicity was ascertained via electronic health records and included the categories Black, White, and other race or ethnicity (including American Indian or Alaska Native, Asian, unknown, and not disclosed). These data were collected to assess for potential confounding.

### Study Variables

The primary exposure was the time frame of the payment incentive and the multifaceted intervention sponsored by BCBSM to support the completion of confirmatory testing among men with low-risk disease. The payment incentive was applied to patients who received a diagnosis between April 1, 2018, and May 30, 2019, constituting the measurement cohort. For these patients, physicians were given 6 months to have patients complete confirmatory testing (ie, the testing window). The payment incentive was the only aspect of the intervention reliant on physicians reaching a performance-related threshold, allowing us to estimate the incentive’s association with completion of confirmatory testing within the context of the broader intervention.

The denominator for the payment incentive was the measurement cohort. The numerator was men who received a diagnosis in that time frame who underwent confirmatory testing (repeat prostate biopsy, MRI, or a genomic test) within 6 months of the initial prostate cancer diagnosis. This testing is in accordance with clinical guidelines.^8^ In addition, men who underwent MRI within 6 months prior to diagnostic biopsy were considered to have undergone confirmatory testing that counted toward the payment incentive benchmark. The incentive’s benchmark, agreed on between BCBSM and MUSIC practices, was that at least 45% of all men with low-risk prostate cancer within the collaborative needed to complete a confirmatory test. To receive the incentive, practices needed to satisfy a second benchmark (ie, 55% completion rate of a patient-reported outcomes survey among men undergoing radical prostatectomy) that was not evaluated in this study, along with the confirmatory testing benchmark. On meeting these benchmarks, enhanced reimbursement on all BCBSM claims covered by commercial preferred provider organization (PPO) plans would be distributed from March 1, 2020, to February 28, 2021. The enhanced reimbursement (ie, higher payment for the same billed services) would apply to all relative value unit–based professional fees, not just those pertaining to prostate cancer. The enhancement’s extent cannot be explicitly stated; however, it was comparable to the Merit-Based Incentive Payment System’s positive payment adjustment (≤4% increase in reimbursement).^21^

### Statistical Analysis

Statistical analysis was performed from October 2024 to June 2025. Demographic variables, disease characteristics, and management approaches were tabulated. With the patient as the unit of analysis, a multilevel logistic regression model was fit to evaluate the association between the payment incentive and confirmatory testing completion. The multilevel model adjusted for our data organization—patients aggregated by practice—and considered practice-level effects as random intercepts. We used a segmented regression to evaluate the association of the payment incentive with confirmatory testing. We modeled a linear background trend, representing the preincentive period. We adjusted regression models for patient variables (age and insurance payer) and practice variables (baseline confirmatory testing completion and payer mix).

We considered January 1, 2017, to March 30, 2018, as the preincentive period. The association between the payment incentive and confirmatory testing completion, assessed among the measurement cohort, was defined as a change in confirmatory testing completion relative to this preincentive period (ie, a deflection in the slope of confirmatory testing completion over time, adjusting for the background trend of the preincentive period). We identified patients who received a diagnosis in the postincentive period, from June 1, 2019, to July 1, 2022. The COVID-19 pandemic took place during the postincentive period. Prior work has considered the beginning of March 2020 to the end of September 2020 as the period where outpatient health care utilization was most depressed due to the pandemic^22,23^; therefore, we excluded men who received a diagnosis between September 1, 2019 (as patients had 6 months to complete confirmatory testing), and September 30, 2020. These men, if included, would have had a portion of their confirmatory testing window fall within the period most affected by the pandemic.

From prior work,^24^ we postulated that the payment incentive’s association with confirmatory testing completion would vary by certain practice-level characteristics, baseline use of confirmatory testing, and the proportion of patients covered by BCBSM. We also formally tested these prespecified hypotheses using interaction terms, which were statistically significant (*P* < .05). First, we sorted practices into groups by their baseline use of confirmatory testing among men with low-risk prostate cancer in 2017. On one hand, practices with a lower baseline would have more room to increase their testing, improving their response to the incentive. On the other hand, a lower baseline may reflect underlying clinician attitudes toward confirmatory testing, such that the incentive does not meaningfully shift behaviors. We defined the bottom 25th percentile (low baseline: <30% of men completing confirmatory testing), IQR (medium baseline: 30%-56% of men completing confirmatory testing), and top 25th percentile (high baseline: >56% of men completing confirmatory testing). Next, given the payment incentive’s reimbursement mechanism, urology practices with more patients covered by BCBSM (and thus, likely more patients with BCBSM commercial PPO plans) stand to benefit the most from the incentive. Among patients covered by BCBSM, we could not distinguish those covered specifically by commercial PPO plans. We sorted practices by their payer mix—the bottom 25th percentile (low BCBSM: <19% of patients covered by BCBSM), IQR (medium BCBSM: 19%-31% of patients covered by BCBSM), and top 25th percentile (high BCBSM: >31% of patients covered by BCBSM).

In a separate analysis, we examined patients with low-volume favorable intermediate-risk disease (grade group 2 with ≤3 positive cores and ≤50% greatest individual core involvement).^25^ We hypothesized that associations of the payment incentive (which applied only to low-risk disease) with confirmatory testing completion would spill over and spur greater confirmatory testing completion among men with favorable-intermediate risk disease.

Last, in a sensitivity analysis, we excluded patients undergoing an MRI prior to their initial diagnostic biopsy. Although these patients were counted toward the payment incentive’s benchmark, clinical guidelines typically only consider confirmatory testing performed after the initial diagnostic biopsy.^8^

Analyses were conducted using Stata, version 18 (StataCorp LLC). Average marginal effects (ie, predicted probability) were calculated using postestimation commands (ie, the margins command). We derived odds ratios (ORs) of the payment incentive from our secondary analyses using a linear combination of parameters (ie, the lincom command). The Jonckheere-Terpstra trend test was used to assess whether there was a statistically significant trend in confirmatory testing over time. All statistical tests were 2-sided and used *P* < .05 to define statistical significance.

## Results

### Cohort Characteristics

**Figure 1.**
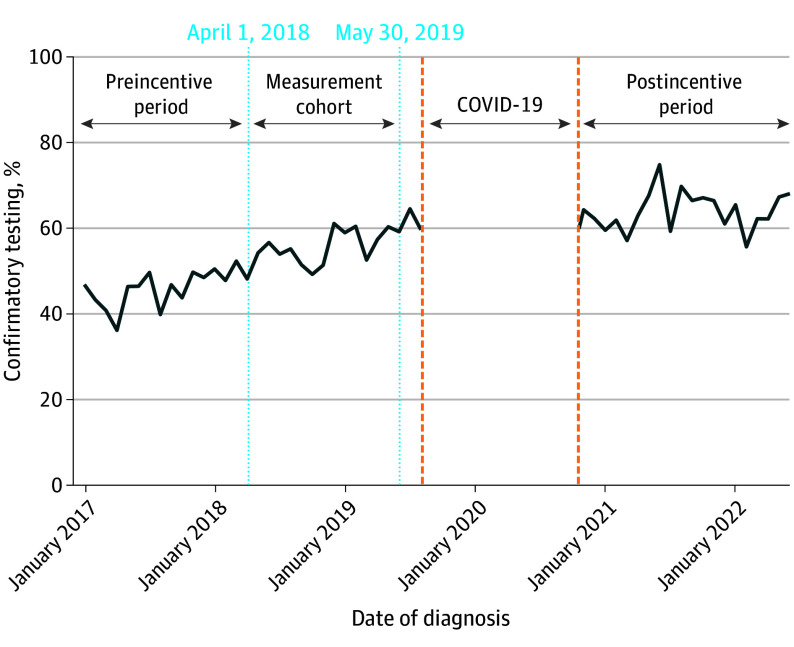
Timeline Among Men With a Diagnosis of Low-Risk Prostate Cancer Men who received a diagnosis between September 1, 2019, and September 30, 2020, were excluded because patients had 6 months to complete confirmatory testing. These men, if included, would have had a portion of their confirmatory testing window fall within the period most affected by the COVID-19 pandemic.

We identified 6609 patients (median age, 65 years [IQR, 60-70 years]; 679 Black patients [10.3%], 5216 White patients [78.9%], and 714 patients of other race and ethnicity [10.8%]) with low-risk prostate cancer (Table). Active surveillance (4818 [72.9%]) was the most common management strategy, followed by radical prostatectomy (806 [12.2%]). Over the study period, the percentage of patients with low-risk prostate cancer undergoing confirmatory testing within 6 months of diagnosis increased from 44.6% (725 of 1625) in 2017 to 64.3% (774 of 1203) in 2022 (Jonckheere-Terpstra trend test; *P* < .001), as illustrated in Figure 1. Among 3665 patients undergoing confirmatory testing, MRI was the preferred modality (1993 [54.4%]) compared with genomic testing (1240 [33.8%]) and repeat prostate biopsy (432 [11.8%]). Of those undergoing MRI, 41.1% of patients (820 of 1993) received it prior to their diagnostic prostate biopsy.

**Table.** Characteristics of Patients With Low-Risk Prostate Cancer

Characteristic	No. (%)
No. of patients	6609
No. of practices	48
Age, median (IQR), y	65 (60-70)
Race and ethnicity	
Black	679 (10.3)
White	5216 (78.9)
Other^a^	714 (10.8)
Diagnosis year	
2017	1625 (24.6)
2018	1604 (24.3)
2019	1092 (16.5)
2020	335 (5.1)
2021	1307 (19.8)
2022	646 (9.8)
Treatment type	
Active surveillance	4818 (72.9)
Androgen deprivation therapy	44 (0.7)
Brachytherapy	91 (1.4)
External beam radiotherapy	196 (3.0)
Radical prostatectomy	806 (12.2)
Watchful waiting	120 (1.8)
No known treatment	483 (7.3)
Other	51 (0.8)
Primary insurance	
BCBSM	1476 (22.3)
BCN	658 (10.0)
Medicare	1846 (27.9)
Medicaid	184 (2.8)
Medicare Advantage–BCBSM/BCN	602 (9.1)
Other commercial	1239 (18.7)
Commercial HMO	390 (5.9)
Other	214 (3.2)

Abbreviations: BCBSM, Blue Cross Blue Shield Michigan; BCN, Blue Care Network; HMO, health maintenance organization.

^a^ Includes American Indian or Alaska Native, Asian, unknown, and those not providing a designation.

### Primary Analysis

**Figure 2.**
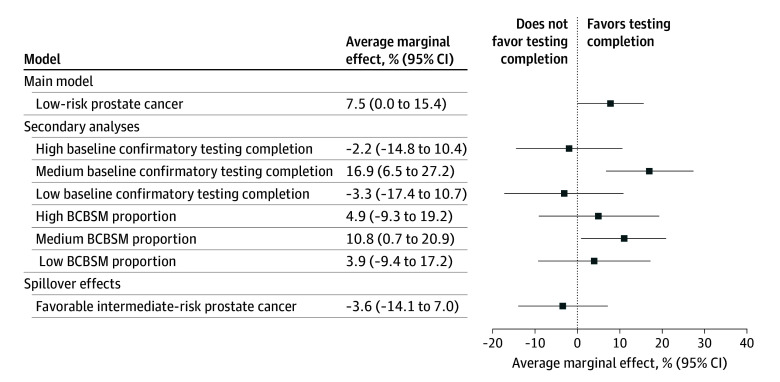
Adjusted Average Marginal Effects of the Payment Incentive—Primary and Secondary Analyses Models adjusted for patient age, insurance payer, diagnosis year, practice-level baseline confirmatory testing, practice-level Blue Cross Blue Shield Michigan (BCBSM) proportion, and fixed effect for years.

During the payment incentive period, men with low-risk prostate cancer tended to undergo more confirmatory testing relative to the preincentive period, but this finding was not statistically significant (OR, 1.43 [95% CI, 0.99-2.09]; *P* = .06). This translated to a 7.5% (95% CI, 0.0%-15.4%) increase in the predicted probability of completing confirmatory testing among the measurement cohort (Figure 2).

### Secondary Analyses

Among men managed by practices in the bottom 25th percentile (OR, 0.83 [95% CI, 0.38-1.83]; *P* = .65) and top 25th percentile (OR, 0.89 [95% CI, 0.45-1.74]; *P* = .73) of baseline confirmatory testing, the payment incentive was not associated with an increase in confirmatory testing completion relative to the preincentive period. However, among those men managed by practices in the IQR of baseline testing, the payment incentive was associated with increased confirmatory testing (OR, 2.10 [95% CI, 1.32-3.24]; *P* < .001), with an average marginal effect of 16.9% (95% CI, 6.5%-27.2%) (Figure 2).

After stratifying practices by proportion of their patient panel covered by BCBSM, we found that the payment incentive was not associated with an increase in confirmatory testing completion among practices in the bottom 25th percentile of BCBSM coverage. We observed an association between the payment incentive and confirmatory testing among practices in the IQR of baseline testing (OR, 1.69 [95% CI, 1.04-2.75]; *P* = .04) but not among practices in the top quartile of BCBSM coverage (OR, 1.27 [95% CI, 0.64-2.51]; *P* = .50) (Figure 2).

### Spillover Effects

In a separate analysis of men with low-volume favorable intermediate-risk prostate cancer (n = 3072), confirmatory testing completion increased from 2017 (49.1% [334 of 681]) to 2022 (68.1% [213 of 313]) (eTable in Supplement 1). Among these patients, relative to the preincentive period, the payment incentive was not associated with an increase in confirmatory testing completion (OR, 0.83 [95% CI, 0.47-1.46]; *P* = .51).

### Sensitivity Analysis

When excluding the 820 patients with low-risk prostate cancer who underwent prebiopsy MRI, the payment incentive was also not associated with increased confirmatory testing (OR, 1.41 [95% CI, 0.95-2.11]; *P* = .09). Additionally, we observed a slightly smaller average marginal effect (7.0% [95% CI, −1.2% to 15.1%) compared with our primary model.

## Discussion

Using a statewide registry, we examined a payment incentive, as part of a broader intervention, to support confirmatory testing completion within 6 months of diagnosis among men with low-risk prostate cancer. The use of confirmatory testing increased during the study period, from 2017 to 2022. The novel payment incentive demonstrated some evidence of increased confirmatory testing completion relative to the preincentive period but did not meet statistical significance.

Despite not reaching statistical significance, the payment incentive to improve confirmatory testing demonstrated a greater effect size than a similarly structured incentive to support the adoption of active surveillance, which was evaluated in prior work.^24^ The distinctions between these programs may offer insight into the broader construction of alternative payment models in prostate cancer. First, unlike active surveillance, a payment incentive for confirmatory testing does not require physicians to constrain spending, instead linking quality with greater utilization (ie, more confirmatory testing). Alternative payment models simultaneously aim to improve quality while incentivizing physicians to reduce global spending, as implied by deferral of treatment in favor of active surveillance, representing a more difficult task in a fee-for-service payment system. These challenges are observed even outside of prostate cancer, as accountable care organizations participating in upside-only (ie, no performance-related penalty) shared savings programs and the Merit-based Incentive Payment System have been largely ineffective at achieving these goals.^19^ Second, completion of confirmatory testing within 6 months of diagnosis has direct, short-term implications for patient management, particularly regarding the choice between active surveillance or immediate treatment.^10^ Third, despite its importance, confirmatory testing is underutilized, as described in our data (ie, 45% of patients with low-risk disease completed confirmatory testing in 2017) and other retrospective series.^9,11^ Consequently, there is considerable room for clinicians to improve their practice. In scenarios where clinicians have relatively little room to improve, ceiling effects may blunt the response to financial incentives.^16^

These ceiling effects may also partly explain the heterogeneity of response to the payment incentive, when stratifying practices by their baseline use of confirmatory testing. The payment incentive was not associated with an increase in confirmatory testing among practices in the top quartile of baseline confirmatory testing (ie, starting at >56% completion rate in 2017), suggesting less room to improve their practice habits. Also, the payment incentive was not associated with increased confirmatory testing among practices in the bottom quartile of baseline confirmatory testing (<30% completion rate in 2017). Here, the payment incentive may not sufficiently motivate certain clinicians and practices to substantially alter their practice patterns. In a prior study of Medicare beneficiaries, practices with the lowest baseline use of active surveillance increased their use of surveillance the least between 2010 and 2014, suggesting a reluctance to change practice habits among certain practices.^26^ Practices in the IQR of baseline confirmatory testing may exist in a “sweet spot,” where we observe the greatest association of the payment incentive with confirmatory testing completion. In these practices, physicians may not only have substantial room to expand their use of confirmatory testing, but also favorable attitudes toward adjusting their practice habits, boosting their sensitivity to the payment incentive and other aspects of the intervention.

Regarding reimbursement policy, our study illustrates strategies to implement disease-specific payment models in prostate cancer and the importance of engaging specialty clinicians to improve quality. Our study suggests that quality-based payment policy may be more effective in contexts where the incentivized physician behaviors are associated with greater health care utilization. In confirmatory testing, quality improvement is not pitted against lower health care utilization, making it easier for some physicians to change practice habits. Similarly, payment policies could be applied to other aspects of active surveillance, such as annual prostate-specific antigen testing or biennial prostate biopsies, as a mechanism to improve quality.

### Limitations

Our study has several limitations. First, efforts to improve confirmatory testing completion stretch beyond the enhanced reimbursement afforded by the payment incentive, which occurred in a relatively focused time frame. Within MUSIC, prior and concurrent initiatives to improve physician and patient education likely improved confirmatory testing even before application of the payment incentive, as evidenced by the increase in testing during the preincentive period. As a result, it is challenging to isolate the associations of the payment incentive with confirmatory testing completion as opposed to other collaborative-wide efforts before and during its application. We have tried to adjust for this by modeling a background trend of confirmatory testing in the preincentive period, before measuring the payment incentive’s association with confirmatory testing completion. Second, we could not capture the magnitude of the payment incentive that an individual urologist might face. Some urologists, such as those in smaller, physician-owned private practices, may immediately notice an incentive’s enhanced reimbursement. However, other urologists, such as those salaried by large academic health systems, are less likely to directly benefit financially, thus dulling their sensitivity to the payment incentive. Third, 3 randomized clinical trials demonstrating the value of MRI prior to biopsy were published in 2018 and 2019.^27,28,29^ Given that patients who underwent MRI prior to biopsy were considered to have completed confirmatory testing, the timing of these publications may have preferentially boosted confirmatory testing rates in the measurement cohort (ie, those who received a diagnosis between April 1, 2018, and May 30, 2019). When excluding these patients in the sensitivity analysis, we observed a slightly smaller effect size. Although prebiopsy MRI may not completely satisfy confirmatory testing by clinical guidelines, recent data suggest that it may be sufficient in select patients electing for active surveillance (ie, grade group 1 with negative MRI findings).^30^ Fourth, the payment incentive’s benchmark requires that patients complete confirmatory testing within 6 months. However, clinical guidelines commonly allow up to 12 months, potentially mitigating the impetus for physicians to change practice habits that are within the range of current recommendations.^8^

## Conclusions

In this cohort study of men with prostate cancer, we found that confirmatory testing steadily improved within MUSIC over the study period. However, the payment incentive specifically was not associated with a robust increase in confirmatory testing completion. Taken together, our results suggest collaboration between payers and physicians has the potential to improve measures of prostate cancer care quality, but also highlight the challenges associated with payment incentives and alternative payment model implementation.

## Notes

Supplement 1. **eTable.** Cohort Characteristics—Favorable Intermediate-Risk Prostate Cancer

Supplement 2.
Data Sharing Statement
