# Supplementary material for: A Payment Incentive to Improve Confirmatory Testing in Men With Prostate Cancer
Source: JAMA Netw Open. 2025 Sep 5;8(9):e2530624. doi: 10.1001/jamanetworkopen.2025.30624 (PMC12413644; doi:10.1001/jamanetworkopen.2025.30624)
Supplement: Supplement 1. — eTable. Cohort Characteristics—Favorable Intermediate-Risk Prostate Cancer [file jamanetwopen-e2530624-s001.pdf]

Supplementary Online Content

Srivastava A, Kaufman SR, Shay A, et al. A payment incentive to improve confirmatory testing in men with prostate cancer. *JAMA Netw Open.* 2025;8(9):e2530624. doi:10.1001/jamanetworkopen.2025.30624

eTable. Cohort Characteristics—Favorable Intermediate-Risk Prostate Cancer

This supplementary material has been provided by the authors to give readers additional information about their work.

© 2025 Srivastava A et al. *JAMA Network Open.*

eTable. Cohort Characteristics—Favorable Intermediate-Risk Prostate Cancer

**Variable**		**No. (%)**
**n (Patients)**		**3,072**
**n (Practices)**		**48**
Diagnosis Year	2017	681 (22.2)
	2018	706 (23.0)
	2019	541 (17.6)
	2020	165 (5.4)
	2021	666 (21.7)
	2022	313 (10.2)
Treatment Type	Active Surveillance	1427 (46.5)
	Androgen Deprivation Therapy	77 (2.5)
	Brachytherapy	95 (3.1)
	External Beam Radiation Therapy	283 (9.2)
	Radical Prostatectomy	746 (24.3)
	Watchful Waiting	37 (1.2)
	No Known Treatment	354 (11.5)
	Other	53 (1.7)
Primary Insurance	BCBSM	594 (19.3)
	BCN	289 (9.4)
	Medicare	967 (31.5)
	Medicaid	95 (3.1)
	Medicare Advantage- BCBSM /BCN	339 (11.0)
	Other Commercial	523 (17.0)
	Commercial-HMO	164 (5.3)
	Other	101 (3.3)
Race	African American	438 (14.3)
	Caucasian	2287 (74.4)
	Other	347 (11.3)

© 2025 Srivastava A et al. *JAMA Network Open.*
