# Supplementary material for: A Payment Incentive to Improve Confirmatory Testing in Men With Prostate Cancer
Source: JAMA Netw Open. 2025 Sep 5;8(9):e2530624. doi: 10.1001/jamanetworkopen.2025.30624 (PMC12413644; doi:10.1001/jamanetworkopen.2025.30624)
Supplement: Supplement 2. — Data Sharing Statement [file jamanetwopen-e2530624-s002.pdf]

Data Sharing Statement

Srivastava. A Payment Incentive to Improve Confirmatory Testing in Men With Prostate Cancer. *JAMA Netw Open*. Published September 05, 2025. doi:10.1001/jamanetworkopen.2025.30624

Data
Data available: No

Additional Information
Explanation for why data not available: The data underlying this article were provided by Blue Cross Blue Shield of Michigan and cannot be shared by the authors of this manuscript under the data use agreement.
